# Genetic characterization and population structure of six brown layer pure lines using microsatellite markers

**DOI:** 10.5713/ajas.17.0870

**Published:** 2018-04-12

**Authors:** Taki Karsli, Murat Soner Balcıoğlu

**Affiliations:** 1 Faculty of Agriculture, Department of Animal Science, Akdeniz University, Antalya 07058, Turkey

**Keywords:** Microsatellite, Genetic Diversity, Population Structure, Pure Chicken Line

## Abstract

**Objective:**

The first stage in both breeding and programs for the conservation of genetic resources are the identification of genetic diversity in the relevant population. The aim of the present study is to identify genetic diversity of six brown layer pure chicken lines (Rhode Island Red [RIRI, RIRII], Barred Rock [BARI, BARII], Columbian Rock [COL], and line 54 [L-54]) with microsatellite markers. Furthermore, the study aims to employ its findings to discuss the possibilities for the conservation and sustainable use of these lines that have been bred as closed populations for a long time.

**Methods:**

In the present study, a total number of 180 samples belonging to RIRI (n = 30), RIRII (n = 30), BARI (n = 30), BARII (n = 30), L-54 (n = 30), and COL (n = 30) lines were genotyped using 22 microsatellite loci. Microsatellite markers are extremely useful tools in the identification of genetic diversity since they are distributed throughout the eukaryotic genome in multitudes, demonstrate co-dominant inheritance and they feature a high rate of polymorphism and repeatability.

**Results:**

In this study, we found all loci to be polymorphic and identified the average number of alleles per locus to be in the range between 4.41 (BARI) and 5.45 (RIRI); the observed heterozygosity to be in the range between 0.31 (RIRII) and 0.50 (BARII); and F_IS_ (inbreeding coefficient) values in the range between 0.16 (L-54) and 0.46 (RIRII). The F_IS_ values obtained in this context points out to a deviation from Hardy-Weinberg equilibrium due to heterozygote deficiency in six different populations. The Neighbour-Joining tree, Factorial Correspondence Analysis and STRUCTURE clustering analyzes showed that six brown layer lines were separated according to their genetic origins.

**Conclusion:**

The results obtained from the study indicate a medium level of genetic diversity, high level inbreeding in chicken lines and high level genetic differentiation between chicken lines.

## INTRODUCTION

Genetic diversity, shaped in the process extending from the past to the present in populations, is of paramount importance for the sustainability of species. In years following the domestication of livestock, the processes of mutation, selection, adaptation, isolation and migration created a large body of genetic diversity in local populations. Nevertheless, intensive breeding processes implemented for the maximisation of various yields among livestock or the preference of high yield breeds to local breeds, have led to decreases in genetic variation in the past 50 years [1,2]. This situation has become even more dramatic in poultry breeding and the number of poultry breeds used in production has decreased due to the breeding system employed in commercial poultry production. The dominant breed in current use for maternal and paternal lines of white layers is the White Leghorn (WL). On the other hand, genetic basis of brown layers comes from the RIR, New Hampshire, Plymouth Rock (PR) and Australorp breeds [3].

The conservation of genetic diversity among livestock and their sustainable use represents one of the extremely interesting and current items on the agenda [4]. Studies on the species of livestock naturally focus on local breeds regarded as an “Animal Genetic Resource”. A similar situation applies also to genetic diversity studies on chickens. Relevant studies concentrate on local chicken breeds. Today, the large part of chicken meat and egg production comes from commercial hybrids especially in developed and developing countries. The pure lines used as a source of such hybrids have been faced with significant decreases in genetic diversity due to their breeding systems and intensive selection process. Studies concerning the identification of genetic diversity of pure lines are unfortunately more limited than local chicken breeds. The identification and conservation of the current genetic diversity of pure lines are necessary not only to provide for today’s demand for production, but also to respond to prospective breeding programmes in the future.

Microsatellite markers are extremely useful tools in the identification of genetic diversity since a multitude are distributed throughout the eukaryotic genome, are present in both introns and exons, demonstrate co-dominant inheritance and feature a high rate of polymorphism and repeatability [2,3,5].

In Turkey, there are six brown layer pure chicken lines (Rhode Island Red [RIRI and RIRII]; Barred Rock [BARI and BARII]; Colombian Rock [COL]; and line 54 [L-54]) and four white layer pure chicken lines (black line, brown line, blue line, maroon line) under Ankara Poultry Research Institute. These populations were imported from Canada in 1995 and have been made subject to selection based on various characteristics by Ankara Poultry Research Institute in Turkey since then. L-54 is synthetic chicken line obtained in 1974 year and it has approximately 15 percent Leghorn blood. Therefore, body weight is lower than other lines and the eggshell colour is quite light brown. Studies undertaken on pure lines resulted in the development of three hybrid materials, i.e. two brown (ATAK, ATAK-S) and one white (ATABEY) layers. RIRI and RIRII are used as sire lines, while BARI and L-54 are used as dam lines to obtain the brown layer hybrids.

The aim of the present study is to identify genetic diversity among six brown layer pure chicken lines using 22 microsatellite loci. Moreover, the study further aims to discuss the possibilities for conservation and sustainable use of these lines bred as closed flocks for a long period, as well as the relationship among the existing chicken lines.

## MATERIALS AND METHODS

### Sample collection and simple sequence repeats genotyping

A total of 180 blood samples including 30 samples from each brown layer pure chicken line in Ankara Poultry Research Institute were taken into tubes with K3 ethylenediaminetetraacetic acid (approximately 1 mL). The blood samples thus collected were stored at −20°C until DNA isolation. DNA was isolated from each line using the protocol reported by Miller et al [6]. DNA concentration was adjusted to 50 ng/μL for polymerase chain reaction (PCR) after DNA isolation. The present study utilised 22 microsatellite loci in total, 17 of which were recommended by FAO [4], for the identification of the genetic structure among brown layer pure chicken lines. The other five loci (ADL0145, LEI0196, LEI0228, MCW0287, MCW0301) were selected from studies on commercial chicken lines [7,8].

### Polymerase chain reaction and fragment analysis

**Figure 1:**
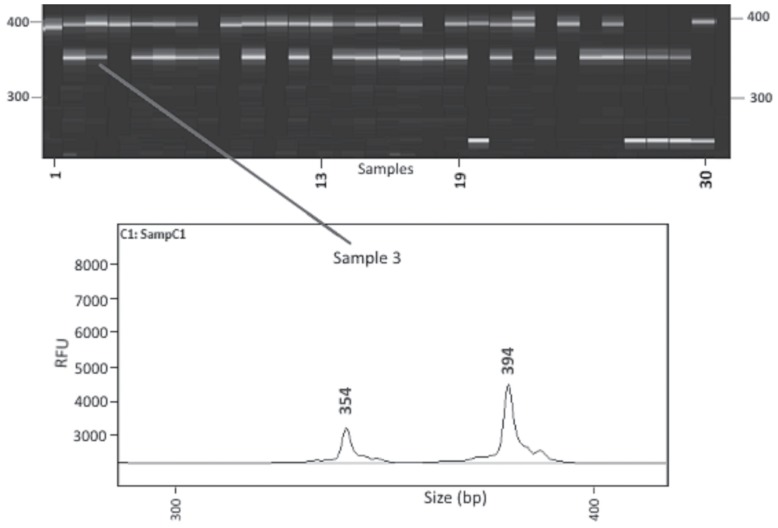
Fragment analyzer genotyping picture on BARII line for LEI0192 locus.

The sizes of PCR products were identified using 96 automated capillary electrophoresis systems (Advanced Analytical Technologies-AATI, Ames, IA, USA). The system is able to differentiate among PCR products up to 2 bp without fluorescent-labelled primers. This system detects size of PCR products by using a sensitive intercalating dye coupled with a powerful LED light source. The gel, inlet buffer, capillary conditioning solution and 35 to 500 bp marker were prepared according to the manufacturer’s instructions using dsDNA 900 Reagent Kit (35 bp/500 bp). Every well of the 96 PCR plate was filled with a 25-μL mixture including 3 μL PCR product and 22 μL 1X dilution buffer and only the last well (H12) was loaded with 25 μL ladder without dilution. After capillary electrophoresis separation, the data was opened and band sizes calculated by using PROSize 2.0 version 1.3.1.1 (Figure 1) (Advanced Analytical Technologies, Inc., USA).

### Data analyses

The identification of genetic diversity within the chicken lines was calculated using allele range (AG), number of alleles (Na) and number of effective alleles (Ne), observed heterozygosity (Ho), and expected heterozygosity (He). Moreover, calculations were undertaken for polymorphism information content (PIC), inbreeding coefficient for the determination of inbreeding among the lines (F_IS_) and genetic differentiation among the lines (F_ST_) the loci under examination. Additionally, factorial correspondence analysis (FCA), Neighbor-Joining tree (NJ) and STRUCTURE clustering analysis were conducted to identify the relationship between lines and individuals.

CONVERT version 1.31 [9] program was used in order to private allele, allele range and frequency. The presence of null alleles in all lines for each locus was tested using ML-NULLFREQ program [10]. Genetic variation parameters (observed and expected heterozygosity, number of allele and effective alleles), genetic identity and genetic distance were calculated using the POPGENE computer program version 1.31 [11]. PIC values with POWERMARKER program [12], inbreeding coefficient using FSTAT v.1.2 [13], pairwise F_ST_ values determined with ARLEQUIN software [14]. The NJ tree was constructed using POPTREE2 [15]. FCA performed by the GENETIX v. 4.05 [16] and Bayesian model-based clustering using STRUCTURE.

Bayesian clustering procedure was implemented in STRUC TURE 2.3 [17]. First, the program was run to assume the number of distinct populations defined as K. The analysis involved an admixture model with correlated allele frequencies. One hundred independent runs with 500,000 Markov chain Monte Carlo iterations and a burn-in of 100,000 steps were performed for 2≤K≤8 (where K is the number of cluster to be tested) to estimate the most likely number of clusters present in the data set. The most probable K was determined using STRUCTURE HARVESTER [18] by calculating the distribution of the ΔK statistic as described by Evanno et al [19]. STRUCTURE PLOT [20] was used to visualize the STRUCTURE output.

## RESULTS

All of the 22 microsatellite loci used in the present study were found to be polymorphic. A total number of 233 alleles were obtained at 22 loci in six chicken lines. The number of alleles per locus was calculated to be 10.59 and the number of effective alleles to be 5.71. The number of alleles observed at loci vary between 4 (MCW0111) and 23 (LEI0228) (Table 1).

In this study, the null allele frequency was detected above 0.2 in only four loci (LEI0196, MCW0020, MCW0287, and MCW0330). The null allele frequency on LEI0196 locus for RIRI, RIRII, BARI, and BARII populations 0.244, 0.329, 0.276, and 0.224, respectively. For MCW0020 locus, RIRII, BARI, and BARII lines as to be 0.319, 0.212, and 0.272, respectively. In RIRI, RIRII, and COL lines, for MCW0287 and MCW0330 loci were calculated as 0.267, 0.255; 0.417, 0.257 and 0.295, 0.242, respectively.

The values for observed and expected heterozygosity in all populations were calculated as 0.42 and 0.79. The F_IS_ and PIC value were determined to be 0.47 and 0.76, respectively. F_IS_ values were found positive in all loci and varied in the range of 0.196 to 0.654. The PIC value is higher than 0.5 in all loci except for MCW0111.

The genetic diversity parameters obtained in six different pure chicken lines are summarised in Table 2. The lowest number of alleles and effective alleles per locus were obtained from BARII (4.41) and RIRII (2.44) lines and the highest numbers from RIRI (5.45, 3.11), respectively.

The F_ST_ value is an indicator of genetic differentiation in sub-populations. Genetic differentiation among populations is high level when the F_ST_ value is higher than 0.25. The lowest pairwise F_ST_ value (0.11) along the lines under examination was found between BARI and BARII lines and the highest pairwise F_ST_ value (0.35) from L-54 and COL lines. The average pairwise F_ST_ value was identified to be 0.29 as obtained for 22 microsatellite markers in all population (Table 3).

Nei’s genetic identity and genetic distance values is shown in Table 4. Among the chicken lines studied, the nearest genetic distance was observed in BARI and BARII lines (0.28) and the longest distance between COL and L-54 lines (1.44). The highest genetic identity was determined between BARI and BARII lines (0.76) and the lowest between COL and L-54 lines (0.24).

**Figure 2:**
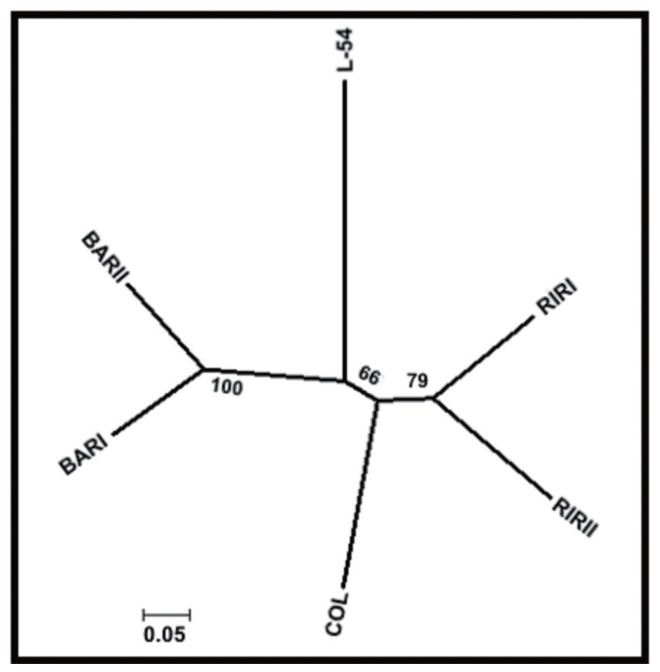
Neighbour Joining tree among the six pure chicken lines based on genetic distance (Nei, 1978). RIRI, Rhode Island Red I; RIRII, Rhode Island Red II; BARI, Barred Rock I; BARII, Barred Rock II; COL, Colombian Rock; L-54, line-54.

**Figure 3:**
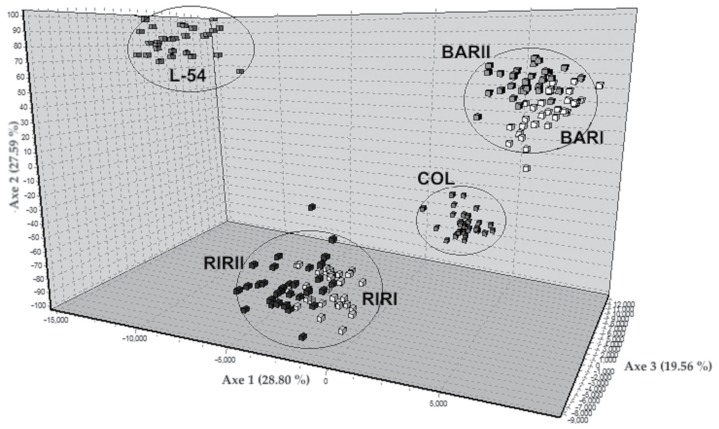
Genetic differentiation illustrated by factorial correspondence analysis (FCA) of the studied six pure chicken lines on 22 microsatellite loci. RIRI, Rhode Island Red I; RIRII, Rhode Island Red II; BARI, Barred Rock I; BARII, Barred Rock II; COL, Colombian Rock; L-54, line-54.

The NJ tree derived from genetic distance is shown in Figure 2. Six layer lines were divided into four groups according to their genetic origins. While BAR and RIR populations clustered two different branches of the tree, COL and L-54 populations formed two separated branches away from them. The FCA was made including all lines and 22 loci using the corresponding allele frequencies (Figure 3). The 75.95% of the total variation was explained by the first three components. The first axis explained 28.80% of the total variation, 27.59% and 19.56% are explained by axis 2 and axis 3, respectively. FCA analysis showed the relationship clearly in six brown layer lines similar to the NJ tree.

**Figure 4:**
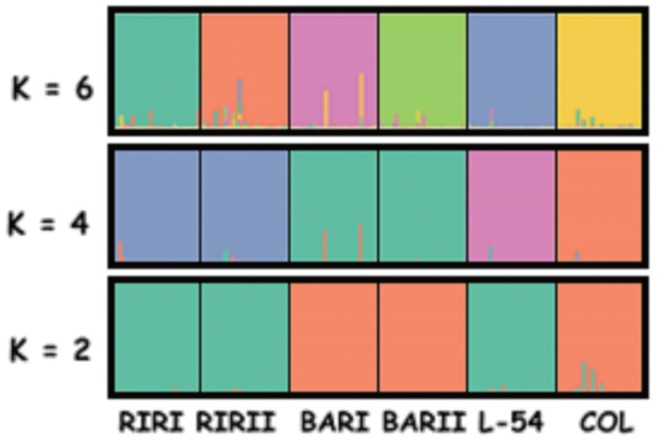
Bayesian cluster analyses of the studied individuals from six brown layer pure lines. Each individual was represented by a vertical bar. The highest ΔK value was obtained for K = 6 of the studied layer pure chicken individuals. RIRI, Rhode Island Red I; RIRII, Rhode Island Red II; BARI, Barred Rock I; BARII, Barred Rock II; COL, Colombian Rock; L-54, line-54.

The differentiation of the lines was also examined on the basis of Bayesian clustering analysis through the use of the Structure programme (Figure 4). When K = 2, L-54 was included in the cluster of RIRI and RIRII, while COL was included in the cluster of BARI and BARII. When K = 4, RIRI and RIRII created one cluster, BARI and BARII one cluster and COL and L-54 two different clusters. When K = 6 where the highest ΔK value was obtained, every line was in a separate cluster with only slight transitions among lines.

## DISCUSSION

The average number of alleles per locus as identified for 22 microsatellite loci in all samples in the present study (10.59) is higher than the values reported by Zanetti et al [21] for four breeds under conservation (8.4); and higher than the values specified by Tadano et al [7], Pham et al [22], and Choi et al [23] for commercial lines (6.7 to 8.4).

The observed heterozygosity value established for the six brown layer pure chicken lines (0.42) is lower than the value reported by Tadano et al [24] for brown layers; by Tadano et al [25] for five PR lines; by Granevitze et al [26] for 5 brown layer and one broiler lines; and by Muchadeyi et al [27] for broiler lines (0.460 to 0.744), but higher than the value reported by Tadano et al [25] for two PR lines; and by Muchadeyi et al [27] for brown and white layer pure lines (0.32 to 0.41).

The average F_IS_ value calculated for all loci in all populations (0.47) is higher than the values reported by Muchadeyi et al [27] for white and brown layer and broiler chicken lines (0.07, 0.02, 0.03, respectively); by Granevitze et al [26] for brown layer lines (0.04); by Rajkumar et al [5] for eight populations including RIR and WL lines (0.13); by Zanetti et al [21] for four populations under preservation (0.42); by Ramadan et al [28] for nine populations including RIR and WL lines (0.05); and by Seo et al [8] for five Korean local chicken lines (0.01).

Comparing the present values with those in the other studies in the literature the number of alleles per locus is higher and the heterozygosity values are at medium levels; but the F_IS_ coefficient is much higher. We observed that the PIC value is higher than 0.50 for all loci except for MCW0111 and that these loci are quite useful for genetic diversity studies. There may be multiple reasons underlying the higher average number of alleles per locus when compared to those in other studies. These reasons can include the six different pure lines used in the present study come from different genetic origins (RIR, PR [BAR and COL]) and have been as closed flocks and subjected to selection for a long period of time. These populations have been selected in Turkey since 1995. According to various resources maintained at Ankara Poultry Research Institute, the lines were bred as closed flocks and selected for various characteristics 45 to 50 years before they entered to Turkey. The selection procedure applied on these lines for 70 years may have led to the emergence of different alleles.

All loci are considered to suffer from a heterozygote deficit arising from inbreeding as a basis for the positive F_IS_ values identified in the present study. If sub-populations are bred as closed flocks in isolation from each other, as is the case in the present study, a deviation from Hardy-Weinberg equilibrium is an expected consequence. However, the F_IS_ values obtained here are much higher than expected. The reason behind the high F_IS_ value is considered to be the small population size.

The number of alleles and effective alleles obtained from pure layer lines in the present study (Table 2) are lower than the number of alleles reported by Rajkumar et al [5] for the Rhode Island Red line and WL lines (3.27 and 4.23, respectively). On the contrary, these figures are higher than the number of alleles reported by Granevitze et al [26] for three brown layer lines (3.04 to 3.44), for four broiler chicken lines (3.74 to 4.79) and for one white layer line (2.96). They are also higher than the number of alleles per locus reported by Tadano et al [7] for RIR-A and RIR-B lines (4.25, 3.28, respectively); the number of alleles reported by Tadano et al [25] for seven PR lines (in the range of 2.70 to 4.20).

The Ho values in the present study varied in the range of 0.31 (RIRII) and 0.50 (BARII); He values in the range of 0.54 (RIRII) and 0.64 (RIRI); and FIS in the range of 0.16 (L-54) and 0.46 (RIRII). All observed heterozygosity values obtained for the lines in the present study are lower than expected heterozygosity values. This finding is an indicator of excess homozygosity in the populations and has led F_IS_ value results to be positive. Similar findings were reported for various pure lines et al [5,26,28]. The F_IS_ value is an indicator of inbreeding within a population and is used to determine the deviation from Hardy-Weinberg equilibrium. At the same time, it is also an important indicator for the designation of conservation priorities for populations.

According to the report by Simon and Buchenauer [29] breeds are not under any danger if the F_IS_ value was below 0.05; they are under potential danger if this value is in the range between 0.05 and 0.25; they were at the minimum level of danger if this value is in the range between 0.15 and 0.25; and the danger reached a critical level if this value is higher than 0.40. Breeds in such a situation needed to be kept under conservation. The F_IS_ values obtained for pure lines utilised in our study indicate that the populations are in serious danger. Specifically, the danger has exceeded the critical level in the RIRII line (0.46) and emergency measures should be taken for this population. The reason for an excess of homozygosity in all populations might be null alleles or subpopulation structure (Wahlund effect) may induce deviation from random mating which leads to higher F_IS_ values. According to Mahammi et al [30], if the null allele frequencies are below 0.20, the effect of null alleles may be acceptably insignificant. In this study, the null allele frequency was above 0.2 in only LEI0196, MCW0020, MCW0287, and MCW0330 loci. This high level of inbreeding is considered to stem from the breeding system or subpopulation structure (Wahlund effect) and small population size rather than the null alleles.

The present study found the average number of alleles per locus and the number of effective alleles to be higher than most of the figures reported by similar studies. In terms of observed and expected heterozygosity values, the population features a medium level of genetic diversity. Nevertheless, the level of inbreeding in the population is higher than that in all relevant studies performed along similar lines in the literature in terms of the FIS values.

The number of private alleles determined in the present study vary between 7 (RIRII, BARII, COL) and 17 (L-54) (Table 2). Private alleles are closely correlated with gene flow and mutation rate. The percentage of private alleles obtained in this study was 24.9% (58/233). This value is much higher than the values reported in previous various studies [7,22,26,27].

The high number of private alleles was expected, because there is a negative correlation between the number of private alleles and migrating individuals in populations. The breeding of the six populations in isolation is the main reason behind the high number of private alleles. Another reason behind this high number of private alleles is the selection procedure performed on these populations for various purposes. Selection performed in different directions for a long period of time may be considered to have led to the emergence of different alleles in the populations.

Tadano et al [24] reported that pairwise FST values on 5 closely related chicken lines produced from Nagoya (NG) chicken breed varied between 0.02 (NG4-NG5) and 0.25 (NG1-NG2), while Tadano et al [25] specified that the same value was in the range between 0.20 (PR2-PR3) and 0.42 (PR4-PR5) along the PR line. Five pure lines obtained from the Nagoya breed, i.e. NG, NG2, NG3, NG4, and NG5, were created in 1903, 1973, 1984, 1992, and 2001, respectively. The seven PR lines were, on the other hand, created in 1960 and 1970s. The length of the applied selection period increases genetic differentiation. Even though BARI and BARII lines and RIRI and RIRII lines have originated from a common genetic origin, the differentiation among them may have stemmed from long years of selection. As stated in previously, these lines have been subject to selection for a period of approximately 70 years.

The reason behind the highest pairwise F_ST_ value (0.35) being identified on COL and L-54 lines is considered to be that the L-54 line carries Leghorn blood at 15%. L-54 is a synthetic line and was created from COL line by adding Leghorn blood at 15%. As can be seen in Table 3, all pairwise F_ST_ values are statistically significant at the level of 0.001. The lines under study are subjected to such a high rate of differentiation as 29% (0.29).

Nei’s genetic distance values ranged from 0.28 (BARI-BARII) to 1.14 (COL-L-54). Muchadeyi et al [27] reported Nei’s genetic distance value among pure lines to be 0.61; Rajkumar et al [5] found the genetic distance value between RIR and two WL populations to be 0.43 and 0.38, respectively; Ramadan et al [28] specified the genetic distance value between RIR and WL populations to be 0.33; and Seo et al [28] determined the genetic distance values on five Korean chicken lines to be in the range between 0.08 and 0.17. FCA analysis showed the relationship clearly in six brown layer lines similar to the NJ tree. The FCA results corroborate the findings based on the Nei’s genetic distances, and clearly separate to four clusters the studied six layer lines.

When K = 6 where the highest ΔK value was obtained, bayesian clustering analysis showed every line was in a separate cluster with only slight transitions among lines. The applied selection and breeding system have lead to high genetic differentiation in chicken lines. In the light of all of these findings, BAR lines may be stated to have diverged from each other later than the other lines. The situation in COL and L-54 lines is considered to have stemmed from the presence of 15% Leghorn blood in the L-54 line.

Consequently, the findings obtained from the study indicate a medium level of genetic diversity and a high level of inbreeding on the chicken lines. Furthermore, genetic differentiation among the populations is also at high levels. Our results show that microsatellite markers are extremely useful in the demonstration of genetic variations even in populations closed for a long period of time and the identification of conservation priorities in populations. For the continued maintenance of activities undertaken at Ankara Poultry Research Institute, various measures are recommended specifically with a view to minimising inbreeding in populations.

## Figures and Tables

**Table 1:** Descriptive statistics for genetic diversity over 22 microsatellite loci in six brown layer pure lines

Locus	N	AR	Na	Ne	Ho	He	PIC	F_IS_
ADL0112	174	120–128	5	4.05	0.40	0.76	0.71	0.48**
ADL0145	171	114–132	8	4.60	0.49	0.79	0.75	0.38*
ADL0268	171	104–128	13	6.92	0.69	0.86	0.84	0.20**
LEI0094	168	245–279	13	5.03	0.41	0.80	0.78	0.50**
LEI0166	175	348–362	8	4.56	0.43	0.78	0.75	0.45**
LEI0192	177	256–402	20	8.52	0.48	0.89	0.87	0.47**
LEI0196	176	174–204	14	7.50	0.30	0.87	0.85	0.65**
LEI0228	175	160–268	23	14.97	0.63	0.94	0.93	0.33**
LEI0234	177	216–314	18	12.14	0.44	0.92	0.91	0.52**
MCW0020	169	175–185	6	3.96	0.33	0.75	0.71	0.57**
MCW0037	174	148–172	9	4.82	0.37	0.80	0.77	0.53**
MCW0067	178	174–186	7	4.29	0.40	0.77	0.74	0.48**
MCW0069	174	152–170	10	4.83	0.55	0.80	0.77	0.31**
MCW0078	175	131–147	8	4.04	0.40	0.76	0.72	0.47**
MCW0081	179	112–130	8	4.43	0.41	0.78	0.74	0.47**
MCW0111	179	98–104	4	2.19	0.22	0.55	0.49	0.59**
MCW0123	170	88–100	7	2.71	0.35	0.63	0.59	0.45**
MCW0183	174	292–334	14	4.11	0.37	0.76	0.73	0.51**
MCW0248	173	215–227	7	4.17	0.45	0.76	0.73	0.41**
MCW0287	166	226–252	10	5.22	0.35	0.81	0.78	0.57**
MCW0301	176	260–278	9	5.40	0.43	0.82	0.79	0.47**
MCW0330	177	258–288	12	7.21	0.44	0.86	0.85	0.50**
Mean	-	-	10.59	5.71	0.42	0.79	0.76	0.47

N, total number of sample used in each loci; AR, allele range; Na, observed number of allele; Ne, effective number of allele; Ho, observed heterozygosity; He, expected heterozygosity; PIC, polymorphism information content; F_IS_, inbreeding coefficient.

* p<0.05,

** p<0.01, deviation from Hardy-Weinberg equilibrium.

**Table 2:** Descriptive statistics for genetic diversity of each pure layer lines over 22 loci (average±standard error)

	MNA	MNE	H_O_	H_E_	PA	F_IS_
RIRI	5.45±1.68	3.11±1.22	0.48±0.21	0.64±0.13	11	0.28**
RIRII	4.95±1.56	2.44±1.02	0.31±0.19	0.54±0.17	7	0.46**
BARI	5.00±1.34	2.85±0.95	0.44±0.20	0.61±0.18	9	0.27**
BARII	4.41±1.30	2.62±0.08	0.50±0.24	0.59±0.13	7	0.18**
COL	4.73±1.20	2.47±0.81	0.37±0.17	0.56±0.16	7	0.35**
L-54	4.86±2.08	2.63±1.12	0.47±0.21	0.56±0.18	17	0.16**

MNA, mean number of alleles per locus; MNE, mean number of effective alleles per locus; H_O_, average observed heterozygosity per locus; H_E_, average expected heterozygosity per locus; PA, number of private alleles and number of private alleles with frequency ≥0.01; F_IS_, inbreeding coefficient; RIRI, Rhode Island Red I; RIRII, Rhode Island Red II; BARI, Barred Rock I; BARII, Barred Rock II; COL, Colombian Rock; L-54, line-54.

** p<0.01, deviation from Hardy-Weinberg equilibrium.

**Table 3:** Genetic differentation (pairwise F_ST_) values among six chicken lines

	RIRI	RIRII	BARI	BARII	L-54
RIRII	0.21***	-	-	-	-
BARI	0.31***	0.35***	-	-	-
BARII	0.32***	0.35***	0.11***	-	-
L-54	0.29***	0.30***	0.30***	0.29***	-
COL	0.26***	0.34***	0.31***	0.32***	0.35***

RIRI, Rhode Island Red I; RIRII, Rhode Island Red II; BARI, Barred Rock I; BARII, Barred Rock II; COL, Colombian Rock; L-54, line-54; F_ST_, genetic differentiation.

All pairwise F_ST_ are significantly different from 0;

*** p<0.001; mean = 0.29.

**Table 4:** Nei’s genetic identity (above diagonal) and genetic distance (below diagonal)

	RIRI	RIRII	BARI	BARII	L-54	COL
RIRI	-	0.59	0.35	0.31	0.29	0.52
RIRII	0.52	-	0.33	0.38	0.35	0.38
BARI	1.06	1.11	-	0.76	0.28	0.40
BARII	1.17	0.98	0.28	-	0.35	0.37
L-54	1.24	1.06	1.26	1.06	-	0.24
COL	0.66	0.97	0.92	0.98	1.44	-

RIRI, Rhode Island Red I; RIRII, Rhode Island Red II; BARI, Barred Rock I; BARII, Barred Rock II; COL, Colombian Rock; L-54, line-54.
